# Isolation, purification, and characterization of antioxidant peptides from fresh mare's milk

**DOI:** 10.1002/fsn3.2292

**Published:** 2021-05-11

**Authors:** Yizaitiguli Waili, Yiming Gahafu, Alimijiang Aobulitalifu, Zhanying Chang, Xiangyun Xie, Gulibahaer Kawuli

**Affiliations:** ^1^ Department of Pharmaceutics and Physical Chemistry College of Pharmacy, Xinjiang Medical University Urumqi China; ^2^ Department of Natural Medicine College of Pharmacy, Xinjiang Medical University Urumqi China

**Keywords:** activity detection, structure identification, whey protein of fresh mare's milk

## Abstract

In this study, the whey protein of fresh mare's milk was used as raw material. The antioxidant peptide liquid XMNDT was extracted from fresh mare's milk solution and purified. The peptide had a molecular weight of 1594.89 kDa and was mainly composed of VAPFPQPVVPYPQR. Antioxidant peptide XMNDT could inhibit the proliferation of A549 lung cancer cells in the G1 phase, accelerate cell apoptosis, increase the activity of SOD and the amount of GSH, and reduce the secretion of MDA. It also exhibited certain antioxidant capacity and free radical scavenging. These data can provide a basis for research on new antioxidant properties by reducing the inflammation caused by aging.

## Graphical abstract

Whey protein of fresh mare's milk; structure identification; and activity detection.

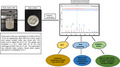

## 1 PREFACE

Fresh mare's milk is a grassland resource with distinctive national characteristics and profound cultural background. The nutritional value of mare's milk is higher than that of other dairy products. From the perspective of nutrition and health care, the effect of mare's milk is better than that of cow's milk. Mare's milk is similar to human milk in terms of chemical composition (Cagali et al., 2014; Claeys et al., 2014; Li et al., 2020). It contains rich nutrients that are involved in human metabolism and functions in adjusting the human body's physiological function, improving immunity, and preventing disease. Unsaturated fatty acid and low‐molecular‐weight fatty acid can prevent hypercholesterolemia and atherosclerosis and exert therapeutic effects on tuberculosis, emphysema, and chronic gastroenteritis symptoms (Kawuli, 2015). With the development of bioactive peptides in milk and dairy products, an increasing number of bioactive peptides from milk sources have been found. They play many roles in the cardiovascular system, immune system, digestive system, and nervous system. The biological activities of these biologically active polypeptides from milk sources have been gradually exploited to produce healthy or functional food to improve human health.

Oxidative stress occurs when the body is subjected to various harmful stimuli in vivo. During oxidative stress, highly active molecules, such as reactive oxygen free radicals (reactive oxygen species) and reactive nitrogen free radicals (reactive nitrogen species) are produced beyond the oxidation degree of oxide removal. Therefore, an imbalance of oxidation system and antioxidant system occurs, leading to tissue damage. The most common diseases induced by elevated levels of oxidative stress are heart disease, cancer, osteoarthritis, rheumatoid arthritis, diabetes, and neurodegenerative problems, such as Alzheimer's disease and Parkinson's disease (Jenner & Olanow, 1996). Antioxidants may slow down the effects of oxidative stress. Milk sources of active peptide have various human metabolic and physiological functions, are easy to digest and absorb, promote immunity, exhibit antibacterial and antiviral activities, and reduce blood pressure and blood lipid levels (Gill et al., 2000; Martínez‐Maqueda et al., 2012; Mohanty et al., 2016; Tsuruki et al., 2005). Meanwhile, it's food safety is very high, which is the most popular research topic and a promising functional factor in the international food industry. Most proteins are digested and absorbed in the form of low peptides, which are absorbed in the form of free amino acids. Peptides are digested faster and are absorbed more than free amino acids, indicating that peptides have higher biological and nutritional values than free amino acids.

A bioactive peptide is a 25‐amino acid protein with different compositions and arrangement from two different to general linear dipeptides. Its ring structure is complex as it is a multifunctional compound derived from protein. Moreover, it is extracted by membrane separation technology, ion‐exchange chromatography or ion gel chromatography, and capillary electrophoresis, reversed‐phase high‐performance liquid chromatography (RP‐HPLC), and time‐of‐flight mass spectrometry were used to further purify and identify the active peptides and to reveal more physiological activities of the peptides. In this study, whey protein of fresh mare's milk was treated with trypsin enzyme solution for 3 hr and then enriched with Sephadex G‐75 to separate proteins by molecular weight. In 3–10 kDa ultrafiltration membranes, the proteins were separated into two groups, namely, A and B. Group B showed the best antioxidant activity. In the B component, the proteins were further purified by RP‐HPLC and then identified using MALDI‐TOF/TOF. The molecular weight of the antioxidant peptide was 1594.89 kDa, and the amino acid composition was VAPFPQPVVPYPQR. Fresh mare's milk antioxidant peptide could inhibit A549 cell proliferation of lung cancer and cause arrest in the G1 phase and increased apoptosis compared with the model group. The antioxidant peptides showed increased glutathione (GSH) content and superoxide dismutase (SOD) activity. The antioxidant activity decreased the secretion of malondialdehyde (MDA), providing the basis for the study of antioxidant peptides in milk sources.

## 2 MATERIALS AND METHODS

### 2.1 Instruments and reagents

#### 2.1.1 Instruments

This study used the following instruments: centrifuge (LG10‐2.4, Beijing Medical Centrifuge Co., Ltd.), freeze dryer (FDU‐2100, Tokyo Chemical Analysis (Sartourius), balance (Beijing Saiduolisi Balance Co., Ltd.), UV–Vis spectrophotometer (UV‐9100D Beijing LabTech Instrument Co., Ltd.), electric thermostatic water bath (HWS‐24, Shanghai Heng Technology Co., Ltd.), decolorization table (WD‐9405A, 61 Beijing Instrument Factory), PHS‐3C laboratory pH meter (Shanghai Ang Yu Environmental Technology Co., Ltd.), MALDI‐TOF/TOF mass spectrometer (5800, Bruker Daltonics Inc., USA), Waters 600 HPLC (Waters, USA), ZEISS Axio Observer Z1 inverted microscope (Zeiss Optical Instruments (Shanghai) International Trade Co., Ltd.), high‐speed refrigerated centrifuge (HC‐3018R, Kdcx Limited by Share Ltd. Zhongjia Branch), ice machine (AF103AS, Shanghai, Delta Industrial Co. Ltd.), ultrasonic cell grinder (JY92‐2D, Ningbo Scientz Biological Polytron Technologies Inc.), and ELISA analyzer (MULTISKAN GO Thermo), ultrafiltration membrane (Millipore 3, 10, and 30 kDa, Shanghai Juncheng Biotechnology Co., Ltd.).

#### 2.1.2 Reagents

This study used the following reagents: fresh mare's milk (Shuixigou Town, Urumqi City, Xinjiang Uyghur Autonomous Region, China), Sephadex G‐50 (Sigma‐Aldrich), trypsin enzyme (activity >250 NFU/mg, Biosharp BioTechnology), 2,2‐diphenyl‐1‐picrylhydrazyl (DPPH, Sigma‐Aldrich), and Sephadex G‐75 (Sigma‐Aldrich). Sodium hydroxide, hydrochloric acid, potassium ferricyanide(III), chloroacetic acid, phosphate buffer, anhydrous alcohol, salicylic acid, hydrogen peroxide, ascorbic acid, ferrous sulfate, copper sulfate, and pyrogallic acid (Analytical Pure, Chinese Pharmaceutical Group Chemical Reagent Co., Ltd.). Antioxidant peptides (95% purity of milk antioxidant peptides based on laboratory findings). Nonsmall‐cell lung cancer cell line A549 was purchased from Boshide Biological. A high‐glucose medium (DMEM) was bought from BI Company. Phosphate‐buffered saline (PBS) was bought from HyClone Company. Fetal bovine serum (FBS) was purchased from Zhejiang Tianhang Biological Co., Ltd., and 0.25% trypsin was purchased from HyClone Company. 2‐Dimethyl sulfoxide (DMSO) was bought from VETEC Company. 4,5‐Diphenyl (MTT) was purchased from Boshide Biology. PE coupled Annexin V apoptosis detection kit was purchased from BD Company; GSH, SOD, and MDA kits were purchased from Nanjing Jiancheng Biological Engineering Institute. Protein quantitative analysis kit was purchased from Thermo Company.

### 2.2 Enzymatic hydrolysis of antioxidant peptides from fresh mare's milk

Fresh mare's milk was centrifuged at 11180×g for 15 min for degreasing. Skim milk and 2 mol/L acetic acid sodium acetate buffer were mixed with 10% acetic acid to adjust the pH to 4.6. The sample was incubated in a 40°C water bath for 20 min and centrifuged at 7155×g for 15 min. The supernatant was collected (whey protein), freeze‐dried, sealed, and stored in a refrigerator at 4°C. The whey protein powder of fresh mare's milk was accurately weighed to 100 mg, diluted to a 30 mg/ml solution, and placed in a 90°C water bath for 10 min, following a previous method (Oftedal et al., 1983; Pikul et al., 2008) for the determination of antioxidant activity. Antioxidant activity was determined with enzyme species, enzyme dosage, enzymolysis time, enzyme, and proportion of whey protein powder to investigate the factors of process conditions and to screen fresh mare's milk for antioxidant peptide enzymes.

### 2.3 Enrichment of antioxidant peptides from fresh mare's milk by ultrafiltration

Ultrafiltration centrifuge tubes with molecular weights of 3, 10, and 30 kDa were selected. Hydrolysis was performed at  1207×g centrifugation for 30 min. Molecular weights of <3, >3, <10, >10, and <30 kDa were used to determine the antioxidant activity of five peptides. The peptides with strong antioxidant activity were frozen at 4°C (Lignitto et al., 2010; Wang et al., 2013).

### 2.4 Peptide purification

The antioxidant peptide with the strongest antioxidant activity was prepared into 20 mg/ml, and 3 ml of the culture fluid was allowed to pass through a 0.45 µm micropore filter. The filtrate was added to a Sephadex g‐75 glycemic gel column (1.5 cm × 60 cm) and then eluted with distilled water at a rate of 0.5 ml/min. The OD value of the eluent was determined at a wavelength of 280 nm. Sephadex G‐75 was used to separate components with the strongest antioxidant value. Approximately 10 mg of the sample was accurately weighed and dissolved in 1 ml of deionized water. The solution was centrifuged at  12880×g for 15 min and filtered through a 0.45 µm membrane. RP‐HPLC purification was then performed using the following conditions: mobile phase: A, 5% acetonitrile + 1‰ TFA (950 ml of deionized water +50 ml of acetonitrile + 1 ml of TFA); mobile phase: B, 90% acetonitrile + 1‰ TFA (450 ml of acetonitrile + 50 ml of deionized water + 500 L of TFA); detection wavelength: 280 nm; flow rate: 1 ml/min; sample size: 50 µl; elution gradient 0–90 (B%).

### 2.5 Structural analysis

A 0.5 µl sample was first placed on the MALDI target plate, dried naturally, placed on 0.5 µl of 4 mg/ml CHCA solution (50% acetonitrile solution containing 0.1% TFA), and dried naturally at room temperature. The samples were analyzed by mass spectrometry using a 5,800 MALDI‐TOF/TOF mass spectrometer. The laser source was 355 nm wavelength Nd: YAG laser, the acceleration voltage was 20 kV, and the data were collected by positive ion mode and automatic data acquisition mode. The enzyme was used to correct the peptide at myoglobin and then corrected by external standard. The primary mass spectrum of the sample ranged from 300 Da to 3,600 Da. MS was performed using reflector positive model with the following parameters: CID (OFF), mass range (700–5,000 Da), focus mass (1,600 Da), fixed laser intensity (4,500) digitizer, and bin size (0.5 ns). Tandem mass spectrometry was performed using the 2 KV positive model with the following parameters: CID (ON), precursor, mass, windows (relative 100 resolution, FWHM), fixed, laser, intensity (5,000), digitizer: bin size (1 ns). The data obtained were retrieved using the MASCOT (V 2.3) software. The search parameters were as follows: NCBI protein database, trypsin digestion, a leaky cutting site, tolerance level for 100 ppm, two MS tolerance at 0.8 Da, no fixed modification, and modified variable settings for methionine oxidation (Motta et al., 2014).

### 2.6 Inhibitory effect of antioxidant peptide from fresh mare's milk on human lung cancer cell line A549

#### 2.6.1 Cell culture and subculture

The A549 cell line was cultured in a high‐glucose DMEM medium containing 10%FBS, penicillin, and streptomycin and cultured at 37°C saturation humidity and 5% CO_2_. The medium was changed once every 2–3 days depending on the cell growth status. After the cells reached 80% confluence (De Simone et al., 2011), they were digested for subculture or for further experiments.

#### 2.6.2 Cell proliferation was detected by MTT assay

The logarithmic growth of tumor cells was evaluated by digesting the cells with 0.25% trypsin and adding 100 µl per hole of a 96‐well cell culture plate, with each hole containing approximately 5 × 10^4^ cells. The plate was incubated at 37°C and 5% CO_2_ humidity. The cancer cells were observed for adherent growth. The supernatant was added with 5, 50, 500, 1,000, and 2,000 µg/ml antioxidant peptide in each hole. The cell culture medium without samples was used as the control group, and each group had five holes. After culturing for 12, 48, and 72 hr, 5 mg/ml of MTT solution was added to each hole, and 20 µl/holes were cultured for 4 hr. The supernatant was discarded, added with 100 µl of DMSO per hole, and gently shaken for 10 min. At 490 nm wavelength, the absorption value of each hole was measured using an enzyme marker, and the average inhibition rate was calculated.

$$\text{Cell inhibition rate =}\;\frac{\left(\text{Control group OD}\;‐\text{Test group OD}\right)}{\text{Control group OD}}\times 100\%$$

#### 2.6.3 Cell cycle was detected by flow cytometry

A549 cells were inoculated in six holes, with each hole containing approximately 1 × 10^6^ cells. The cells were cultured to logarithmic phase and then washed with culture medium and PBS. Antioxidant peptide was added to the low‐, middle‐, and high‐dose groups. Antioxidant peptide was also added to the control group. The cells were allowed to grow after 48 hr of trypsin treatment. Approximately 0.5 ml of cells were collected per hole, digested for 1–2 min, and added with 2 ml of medium. The single‐cell suspension was added into a centrifuge tube, centrifuged at  134×g for 5 min. The supernatant was discarded, and the cells were washed twice with 1 ml of PBS. The supernatant was removed, and 1 ml of PBS was added to the heavy suspension and uniformly mixed. Approximately 2 ml of 70% ethanol precooled at 4°C overnight was added to the cells and centrifuged. The supernatant was discarded, and the cells were washed with PBS and then centrifuged. Approximately 500 µl of PI solution was added to stain for 30 min at room temperature. The cell suspension was subjected to flow cytometry. Cell cycle was measured, and the percentage of cells in the G1, S, and G2 phases was observed.

#### 2.6.4 Apoptosis was detected by flow cytometry

The A549 cells were inoculated in six cell culture plates, with each hole containing approximately 1 × 10^5^ cells. The cells were allowed to grow at 80% confluency, and antioxidant peptides were added to the control group, low‐dose group, medium‐dose group, and high‐dose group. The cells were then cultured for 48 hr. Approximately 0.5 ml of cells per hole were collected and digested trypsin without EDTA for 1–2 min and then added with 2 ml of medium. The single‐cell suspension was placed into a centrifuge tube, centrifuged at  134×g for 5 min, and added with 1 ml of PBS. The supernatant was discarded, and then the cells were subjected to centrifugal washing twice. Cells were added with 100 μl of 1× binding buffer. The solution was weighed in cell suspension with 5 μl 7‐AAD and 5 μl of Annexin V solution. The solution was mixed and incubated at room temperature under dark conditions after 15 min of incubation with 400 μl of 1× binding buffer solution. The solution was then evaluated for apoptosis using flow cytometry (Coyle et al., 2008). The experiment was repeated three times.

### 2.7 Antioxidant peptide of fresh mare's milk relieves oxidative stress

#### 2.7.1 Method for establishing cell hypoxia reoxygenation model

The physical model of oxygen (mixed gas, preculture method) was constructed to develop good cell culture dishes in a sterile airtight container. The cells were subjected to air inlet Walter 95%N_2_ + 5%CO_2_ mixed gas outlet. The outflow of the container was sealed to maintain oxygen for 6 hr. The cells were then placed back into the 37°C and 5% CO_2_ incubator, reoxygenated, and cultured for 3 hr.

#### 2.7.2 Experimental grouping

The good growth of lung cancer cells with 2 × 10^5^ cell density were seeded in 60 mm dishes, cultured for 24 hr, and then randomly divided into five groups: blank control group, model group, low‐dose peptide group, medium‐dose peptide group, and high‐dose peptide group. The model and blank control groups were only added with 5% basal culture medium. After 48 hr, the blank control group was allowed to continuously grow normally. The low‐dose peptide group, middle‐dose peptide group, high‐dose peptide group, and model group were treated with hypoxia for 6 hr and reoxygenated for 3 hr. The centrifuged supernatant was used for the experiment. The contents of GSH, SOD, and MDA were calculated according to the formula given by each reagent box. In each experiment, three holes in the experimental group were used, and the experiment was repeated three times.

#### 2.7.3 GSH detection

Approximately 2 × 10^5^ cells were cultured in 60 mm Petri dish. The growth of adherent cells reached 80%. The blank model group, low‐dose group, middle‐dose group, and high‐dose group of antioxidant peptides were cultured for 48 hr. The antioxidant activity of peptide intervention was evaluated after 48 hr, and hypoxia was induced for 6 hr. The cells were reoxygenated for 3 hr. The entire medium of each hole was then collected and centrifuged. The supernatant was separated and cryopreserved at −80°C. The cells were digested using 0.25% trypsin. The collected cells were washed with PBS 1–2 times and collected by low‐speed centrifugation. The cells were then suspended by adding 0.3–0.5 ml of 0.1 M isotonic PBS buffer cells (pH 7.4). The cells were then subjected to ultrasonic or manual grinding to break up the cells. Approximately 0.1 ml of broken cell suspension was added to 0.1 ml of precipitate and uniformly mixed. The suspension was centrifuged at  ​1640×g for 10 min, and the supernatant was obtained for measurement. Minimal GSH test was performed according to the kit instructions by using the microplate method. The cells were suspended by cell lysate and placed on ice for 5 min. The cells were then centrifuged for  1640×g at 4°C for 10 min. Approximately 1 ml of the supernatant was used for color reaction. Absorbance was detected at 420 nm with an enzyme analyzer (Cai et al., 2002). GSH content was calculated according to the following formula:

$$\text{GSH concentration}=\frac{\left(\text{test OD}‐\text{blank OD}\right)}{\text{standard OD}‐\text{blank OD}}\times \text{Standard concentration}\times \text{Dilution factor}÷\text{Proteincon centration}$$

#### 2.7.4 SOD testing

Approximately 2 × 10^5^ cells were cultured in a 60 mm Petri dish. The adherent cells were allowed to grow until 80% confluency. The blank model group, low‐dose group, middle‐dose group, and high‐dose group of antioxidant peptides were cultured for 48 hr. Antioxidant activity of peptide intervention was determined after 48 hr. Hypoxia was induced for 6 hr, and the cells were reoxygenated for 3 hr. The liquid was absorbed, and 0.25% of pancreatic enzymes was used for digestion for 2–3 min. DMEM medium was added to terminate the digestion. The cell suspension was then loaded into 2 ml EP tube and centrifuged at 1,000 rpm for 10 min. The supernatant was discarded, and the precipitated cells were added with 1 ml of PBS, gently blown, and then centrifuged at 1,000 rpm for 10 min. The supernatant was discarded, and the cells were manually ground ultrasonically for measurement. SOD was tested according to the manufacturer's instructions. WST‐1 method was used to detect absorbance at 450 nm with an enzyme analyzer (Wang et al., 2016). SOD activity was calculated according to the following formula:

$$\text{SOD vitality}=\frac{\text{SOD Inhibitory rate}(\%)}{50\%}\times \text{Reaction system Dilution}\times \text{Dilution times prior to sample testion}$$

#### 2.7.5 MDA testing

Approximately 2 × 10^5^ of cells were cultured in a 60 mm Petri dish. The adherent cells were allowed to grow until 80% confluency. Blank model group, low‐dose group, middle‐dose group, and high‐dose group of antioxidant peptides were cultured for 48 hr. The antioxidant activity of peptide intervention was determined after 48 hr. Hypoxia was induced for 6 hr, and the cells were reoxygenated for 3 hr. The cells were collected, added with 0.5 ml of agent five extract, and mixed for 2 min. The cells were broken and added to 0.1 and 1.5 ml centrifuge tubes. ELISA was performed according to the kit instructions. Absorbance at 532 nm was then determined (Xingxing et al., 2016). Sample MDA was calculated according to the following formula:

$$\text{MDA}=\frac{\left(\text{Test group OD}‐\text{Blank group OD}\right)}{\left(\text{Standard group OD}‐\text{Blank group OD}\right)}\times \text{Standard concentration}÷\text{Protein concentration}$$

### 2.8 Statistical analysis

The SPPSS19.0 statistical software was used to analyze the measurement data to represent the group and perform the *t* test. When *p* < .05, the difference was statistically significant. All tests were repeated three times.

## 3 RESULTS

### 3.1 Ultrafiltration enrichment of antioxidant peptides from fresh mare's milk

The whey protein of fresh mare's milk was digested with trypsin enzyme for 3 hr and then subjected to high‐speed centrifugation to remove insoluble enzyme solution. The ultrafiltration membrane was then enriched for 5 peptides with different molecular weight ranges, namely, <3, >3, <10, >10, and <30 kDa. Different molecular weight peptides were simultaneously determined. Reduction capacity was determined to assess hydroxyl radical scavenging ability. DPPH free radical scavenging ability was used as the index. Antioxidant peptides with a molecular weight of >10 kDa showed the strongest antioxidant activity. Therefore, they were selected for the following experiment. The results are shown in Table 1.

**TABLE 1:** Detection of antioxidant activity of antioxidant peptides in fresh mare's milk

Molecular weight range	<3 KDa	>3 KDa	<10 KDa	>10 KDa	<30 KDa
Reducing power (%)	23.67 ± 0.0031	49.45 ± 0.0021	52.44 ± 0.0012	62.19 ± 0.0034	32.38 ± 0.0020
Hydroxyl radical scavenging activity (%)	7.12 ± 0.1245	23.18 ± 0.1221	3.45 ± 0.2317	31.6 ± 0.1631	14.01 ± 0.1374
DPPH scavenging capacity (%)	61.71 ± 0.3648	56.94 ± 0.2567	4.46 ± 0.3217	69.6 ± 0.2313	69.02 ± 0.2167

### 3.2 Separation and purification of antioxidant peptides from fresh mare's milk by Sephadex G‐75 gel chromatography

**FIGURE 1:**
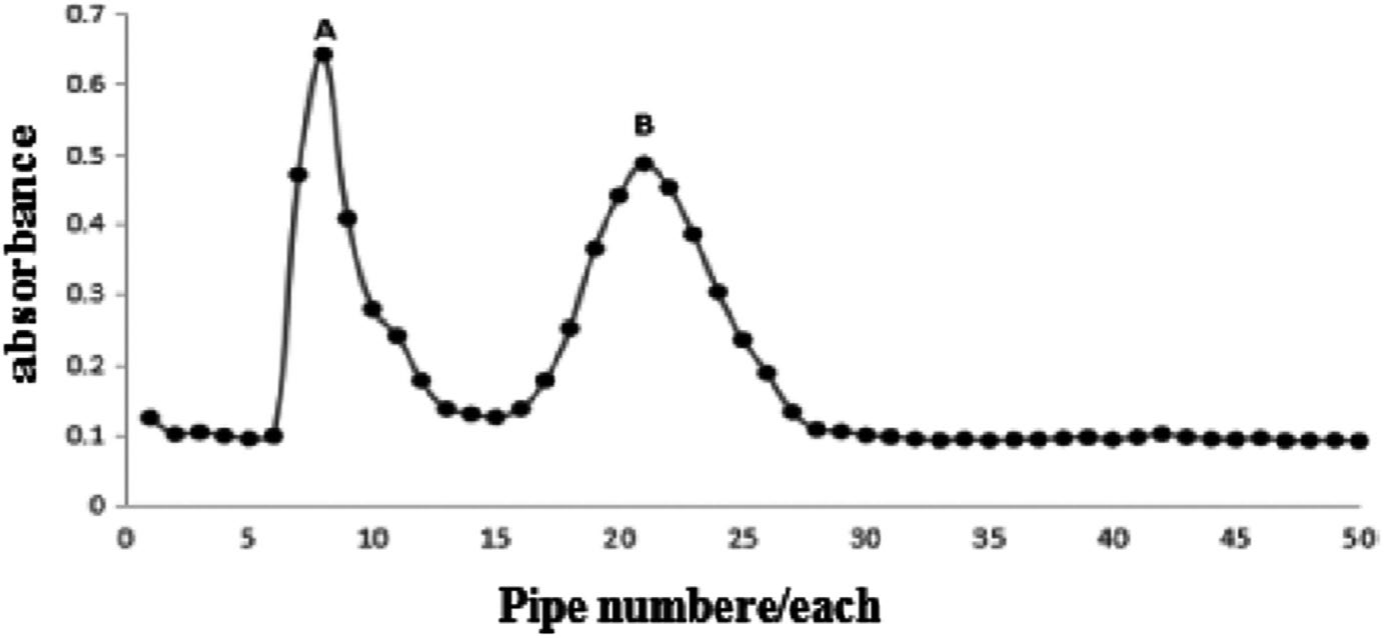
Sephadex G‐75 gel chromatography

The molecular weight of antioxidant peptide >10 kDa was accurately determined. Approximately 5 mg/ml of the solution was filtered through a 0.45 µm microporous membrane. The filtrate sample in the Sephadex G‐75 column was obtained and added to 50 tubes. Two elution peaks of A and B were isolated, and their antioxidant activity and reducing power were determined. Group B showed higher reductive ability, hydroxyl radical scavenging ability and DPPH scavenging ability than group A, were 87.38%, 39.50%, and 75.57%, respectively. The results are shown in Figure 1.

### 3.3 RP‐HPLC isolation and purification

**FIGURE 2:**
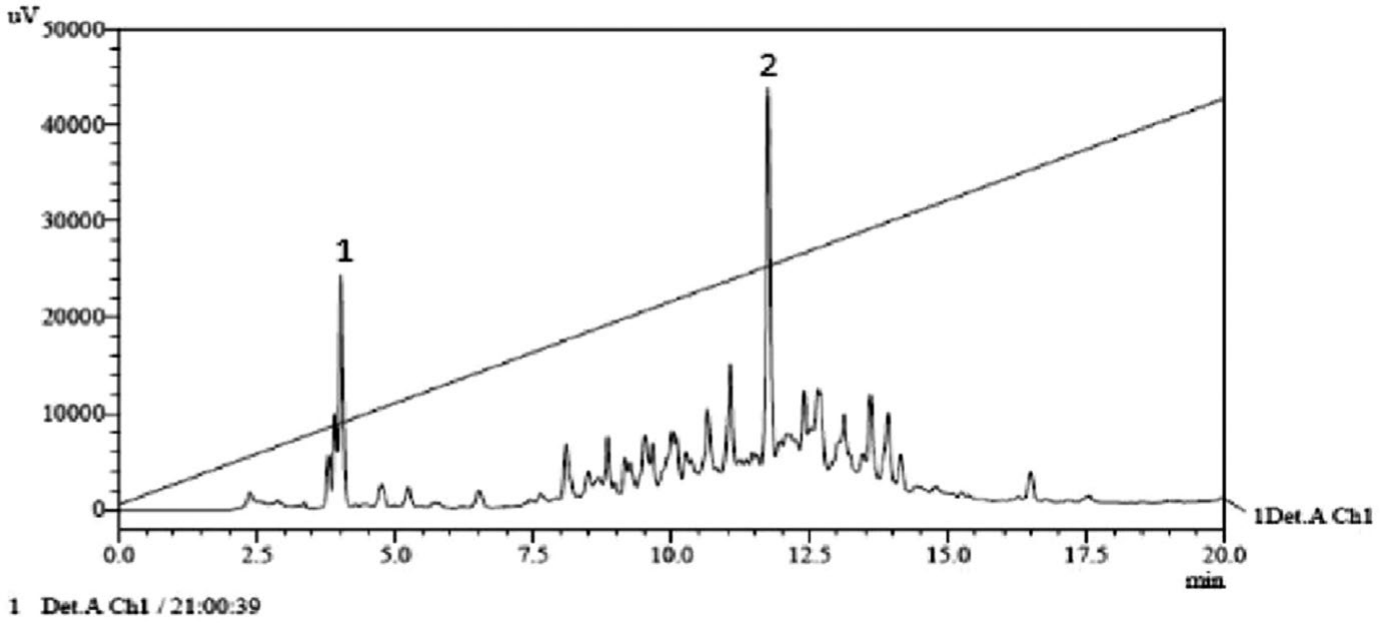
Map of Semi preparative RP‐HPLC

Component B was subjected to RP‐HPLC separation and purification (Figure 2). For the gradient collection, a total of 63 pipe components were freeze dried and detected for antioxidant activity. Among them, two components had strong antioxidant activity, and the activity of component 2 was higher than that of component 1. The results are shown in Figure 2 and Table 2.

**TABLE 2:** The antioxidant activity of 1, 2 fraction was obtained by semi ‐ Preparation of RP‐HPLC ($\bar{X}\pm S$, *n* = 3)

Component	1	2
Reducing power (%)	62.22 ± 0.0012	88.75 ± 0.0026
Hydroxyl radical scavenging activity (%)	27.34 ± 0.1321	49.41 ± 0.1452
DPPH scavenging capacity (%)	62.01 ± 0.4538	76.16 ± 0.4936

### 3.4 MALDI‐TOF/TOF analysis

**FIGURE 3:**
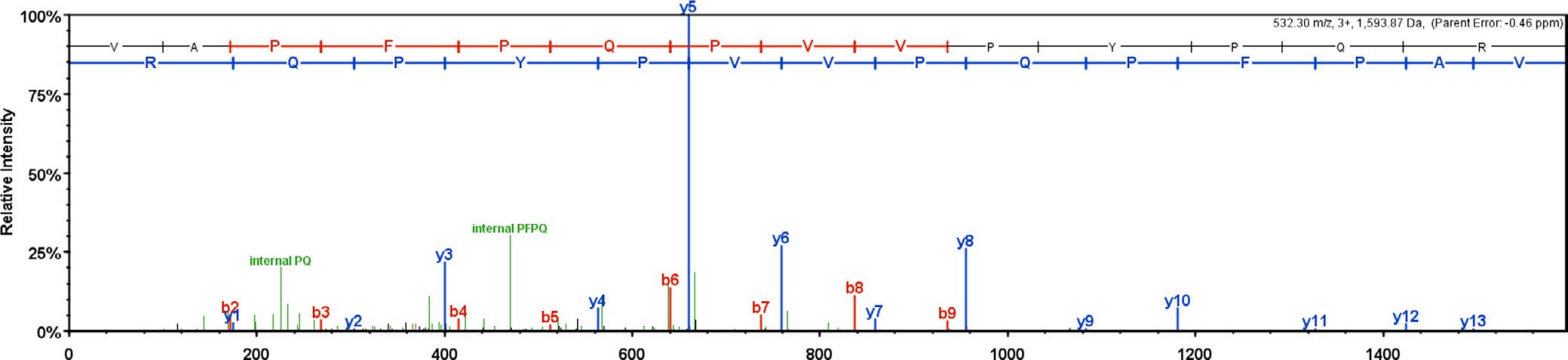
Component MALDI‐TOF/TOF mass spectrometry

The two components were analyzed by MALDI‐TOF/TOF. Figure 3 shows the spectrum, combined with the amino acid composition analysis results. The two components with relative peptide molecular mass of 1594.89 kDa showed that the enzymatic peptide group in whey protein of mare's milk contained VAPFPQPVVPYPQR. The results are shown in Figure 3.

### 3.5 Antioxidant peptide inhibits the proliferation of A549 lung cancer cells

A total of 24 and 48 antioxidant peptides were observed for 72 and 96 hr. The added value of A549 cells were determined and compared with the control group. Different concentrations of antioxidant peptides were added to inhibit A549 cells (*p* < .01). The difference was statistically significant. The results showed that antioxidant peptide in 500–2,000 µg/ml concentration range inhibited the growth of A549 cells. The inhibition rate increased along with the increase of antioxidant peptide concentration and treatment time. The difference was statistically significant in each dose group and control group. The results are shown in Table 3.

**TABLE 3:** Effects of different concentrations of antioxidant peptides on the proliferation of A549 ($\bar{X}\pm S$, *n* = 6)

Group	Inhibition rate of 24 hr/%	Inhibition rate of 48 hr/%	Inhibition rate of 72 hr/%
The control group	0	0	0
5 μg/mL	10.91 ± 2.7431	13.89 ± 1.7810	18.54 ± 1.7523
50 μg/mL	18.23 ± 1.1610	26.25 ± 1.7463	28.68 ± 1.6352
500 μg/mL	27.44 ± 1.5241	38.44 ± 1.6541**	41.77 ± 1.2582**
1,000 μg/mL	33.22 ± 1.7421*	47.24 ± 1.3546**	50.67 ± 1.8643**,***
2,000 μg/mL	38.27 ± 1.6245**	57.19 ± 1.8521**,***	64.84 ± 1.9124**,***

Note

Compared with the control group, *p < .05, **p < .01. Compare with 24 hr, ***p < .05.

### 3.6 Effects of antioxidant peptide on the cell cycle of A549 lung cancer cells

**FIGURE 4:**
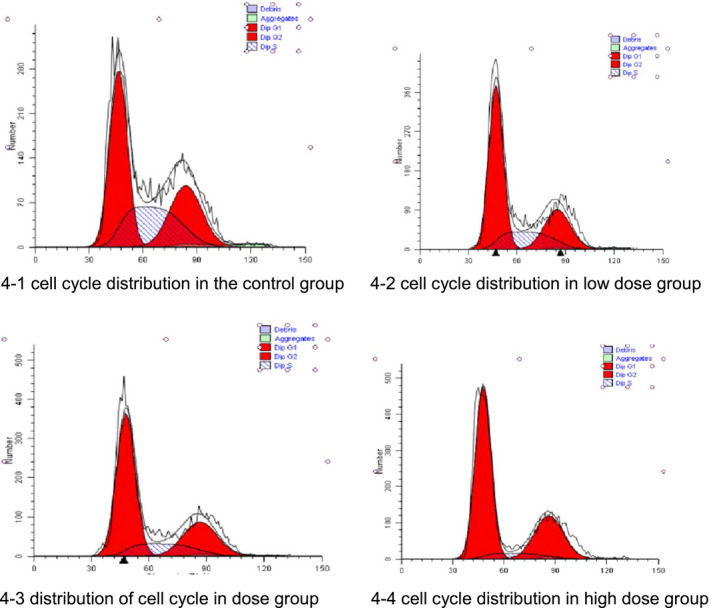
(1) cell cycle distribution in the control group (2) cell cycle distribution in low‐dose group (3) distribution of cell cycle in dose group (4) cell cycle distribution in high‐dose group

As shown in Table 3, compared with the control group, the antioxidant peptide group showed significantly increased ratio of G1 phase cells (*p* < .05). However, the antioxidant peptide group showed significantly decreased S phase cells compared with the control group (*p* < .05). Antioxidant peptides could block cell proliferation in the S phase (Table 4, Figure 41–4).

**TABLE 4:** Cell cycle distribution ($\bar{X}\pm S$, *n* = 3)

Group	G1 stage	S stage	G2 stage
Control group	44.7567 ± 1.3054	29.3833 ± 1.5783	25.8600 ± 1.5649
Low‐dose group	54.5233 ± 1.3963	20.4433 ± 1.48138	25.0333 ± 1.9765
Medium‐dose group	58.6500 ± 1.6254*	16.0900 ± 1.8521*	25.2600 ± 1.8421
High‐dose group	64.69 ± 0.7017**	6.8367 ± 0.0651**	28.4633 ± 0.6786

Note

Compared with the control group, *p* < .05*, *p* < .01**

### 3.7 Apoptosis of A549 lung cancer cells induced by antioxidant peptide

The results showed that with the prolongation of treatment time, A549 cell apoptosis was induced by antioxidant peptides. Compared with the control group, the low‐dose and high‐dose groups reached 19.13% and 24.33% apoptosis rate, respectively, which were significantly different (*p* < .01). Thus, antioxidant peptides have certain cytotoxic effect on A549 lung cancer cells. The results are shown in Table 5 and Figure 51–4.

**TABLE 5:** Effects of antioxidant peptides on apoptosis ($\bar{X}\pm S$, *n* = 6)

Group	Early apoptotic cells/%
Control group	5.9667 ± 0.5033
Low‐dose group	11.1333 ± 0.4509
Medium‐dose group	19.1333 ± 0.7024*
High‐dose group	24.3333 ± 0.9237*

Note

Compared with the control group, *p < .01.

**FIGURE 5:**
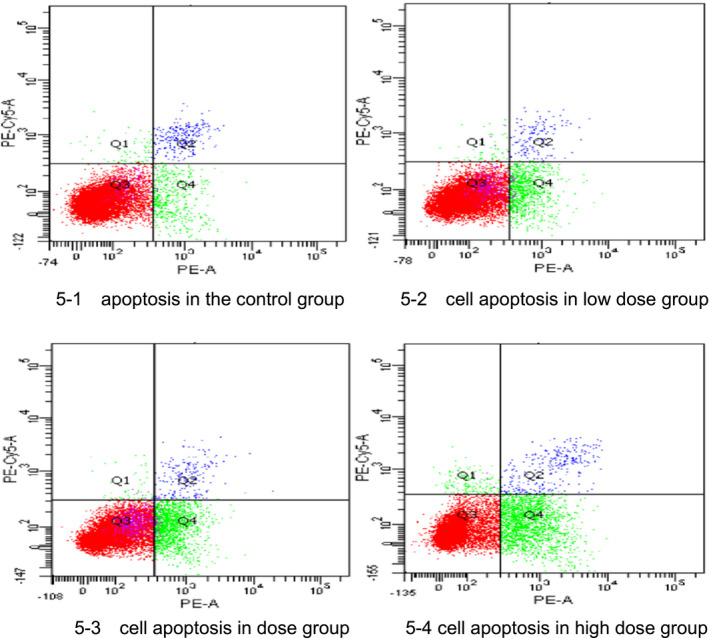
(1) apoptosis in the control group (2) cell apoptosis in low‐dose group (3) cell apoptosis in dose group (4) cell apoptosis in high‐dose group

### 3.8 Oxidative stress index results

Compared with the blank control group, the model group showed significantly decreased secretion of GSH and SOD and significantly increased secretion of MDA (*p* < .01). Compared with the normal control group, the high‐dose group showed significantly increased amount of GSH and SOD secreted by antioxidant peptide and significantly decreased secretion of MDA (*p* < .01). Thus, the antioxidant peptide of fresh mare's milk decreased the toxic substances produced in vivo and had a certain effect of alleviating oxidative stress. The results are shown in Table 6.

**TABLE 6:** Effects of antioxidant peptides on GSH content, SOD and MDA activity in A549 cells ($\bar{X}\pm S$, *n* = 6)

Group	GSH (µmol/gprot)	SOD/(U/mg)	MDA/(nmol/mg)
Control group	13.6555 ± 1.2448	81.3314 ± 0.3387	1.8491 ± 0.7925***
Model group	7.69 ± 0.6935	76.7768 ± 0.1608	9.2456 ± 1.0998*
Low‐dose group	12.9367 ± 1.1561	78.0782 ± 0.1897	5.2853 ± 0.2643
Medium‐dose group	22.2258 ± 1.4505*^,^***	90.4405 ± 0.1629*^,^***	5.2874 ± 0.6653
High‐dose group	28.7713 ± 1.2458**^,^***	95.4826 ± 0.1629**^,^***	2.3784 ± 0.6650***

Note

Compared with blank group, *p < .05, **p < .01. Compared with model group, ***p < .05.

## 4 DISCUSSION

Antioxidant peptide is a kind of peptide with antioxidant activity. There are naturally occurring antioxidant peptides in organisms, mainly carnosine and glutathione (Fraternale et al., 2016; Olivares et al., 2012). However, most antioxidant peptides are isolated from various proteins (Wang et al., 2014). Antioxidant peptides have become the focus of research because of their unique physiological functions and physicochemical properties. Antioxidant peptides can eliminate excess free radicals in organisms and play an important role in promoting the biological activity of antioxidant enzymes and membrane lipid peroxidation and protecting against cell injury (Xianying et al., 2016). The antiaging and oxidative stress characteristics of antioxidant peptide are widely researched.

This study extracted antioxidant peptides by enzymatic hydrolysis and ultrafiltration and performed enrichment of whey protein of fresh mare's milk. Antioxidant activity was evaluated. The hydroxyl radical scavenging abilities of the five peptides were, from high to low, >10 KD, <30 KD, <3 KD, b> 3 KD, and <10 KD. Peptides with molecular weight >10 kDa showed the best antioxidant activity. This is similar to the previous report (Verma et al., 2017; Verma et al., 2018). Two components were separated by Sephadex G‐75. The scavenging rate of free radicals in A components was 23.18%, and that of B components was 39.50%. The scavenging ability of free radicals in B components was obviously larger than that of A components. The antioxidant active components of whey proteases were mainly concentrated in B components. The B components were further purified with RP‐HPLC. A total of 63 fractions were collected. The two components showed the strongest activity and were subjected to MALDI‐TOF/TOF for structural analysis. The molecular weight of small peptide components was 1594.89 kDa, and the amino acid composition was VAPFPQPVVPYPQR.

In China, lung cancer (Sun et al., 2020) ranks first in the list of cancer deaths. The occurrence and development of lung cancer are caused by many factors, which may involve the loss of apoptosis function. The cells then become malignant and proliferate. This feature is one of the important factors of cancer occurrence and development. Therefore, this study evaluated the value‐added role of antioxidant peptides by MTT detection of A549 cells. The experimental results showed that compared with the control group, the antioxidant peptide inhibited lung cancer A549 cell growth in a dose‐ and time‐dependent manner. The concentration of antioxidant peptide increased as treatment time increased, and the difference was statistically significant in each dose between the two groups (*p* < .05). Furthermore, flow cytometry revealed that antioxidant peptides could block the cell cycle of A549 cells in the G1 phase. The number of cells increased with decreased concentration of antioxidant peptide. With the prolongation of injection, A549 cell apoptosis was induced by antioxidant peptides. Compared with the control group, the low‐dose and high‐dose groups showed 19.13% and 24.33% apoptosis rates, respectively, which were statistically significant (*p* < .01). Thus, antioxidant peptide has certain cytotoxic effect on A549 lung cancer cells and has certain inhibitory effect on lung cancer cell growth.

Oxidative stress is one of the major causes of cell death. Many foreign chemicals may cause oxidative damage when they enter an organism (Kazan & Kalaipandian, 2019). GSH peroxidase (GSH‐Px) and total SOD (T‐SOD) are important enzymes in the antioxidant defense system of cells and play an important role in the dynamic balance of oxidation and antioxidation in the body. GSH‐Px can promote the reaction of H_2_O_2_ with GSH to produce water and oxidized glutathione (GSSG). T‐SOD can scavenge superoxide anion radicals and prevent cell damage. As an important nonenzymatic antioxidant, GSH plays an important role in protecting cells from oxidative stress damage (Wan and Stockwell, 2016). Lipid peroxidation is common when oxygen free radicals are excessive, and MDA is the most important product of lipid peroxidation and is an important marker of oxidative stress in cells.

In this study, we detected GSH, SOD, and MDA and found that antioxidant peptide induced lung cancer cells growth at 48 hr. Each drug group content in the culture supernatant of GSH cells was significantly increased. SOD activity was significantly increased, whereas MDA content was decreased significantly. The protective effect of antioxidant peptides in whey protein of fresh mare's milk on oxidative stress injury was preliminarily determined. These results show that the antioxidant peptide can regulate the intracellular antioxidant system, inhibit the free radicals produced during oxidative stress, and restore the oxidative damage to a certain extent. However, the effect of antioxidant peptides on the signaling pathway in lung cancer cells and the mechanism of apoptosis induced by lung cancer have not been fully elucidated. Thus, further research is needed.

## CONFLICT OF INTEREST

The authors declare that they have no competing interests.

## ETHICAL APPROVAL

This study does not involve any human or animal testing.
